# Efficacy of CO_2_ laser with computerized scanner in the treatment of recalcitrant plantar warts: A retrospective case series

**DOI:** 10.1016/j.jdin.2024.12.003

**Published:** 2025-01-14

**Authors:** Ko Kagoyama, Teruhiko Makino, Tadamichi Shimizu

**Affiliations:** Department of Dermatology, Faculty of Medicine, Academic Assembly, University of Toyama, Toyama, Japan

**Keywords:** carbon dioxide laser with a computerized scanner, CO2, intractable, plantar warts, verruca vulgaris

*To the Editor:* Plantar warts, which are caused by infection with human papillomavirus, are occasionally resistant to various therapies, such as topical application of salicylic acid or cryotherapy.^1^ We previously reported a small-scale study that demonstrated the efficacy of a treatment using a CO_2_ laser with a computerized scanner for refractory plantar warts.^2^ In this study, we retrospectively evaluated the efficacy and safety of this treatment in 121 patients with recalcitrant plantar warts. This study was performed under conditions that complied with the principles of the Declaration of Helsinki and was approved by the Medical Ethics Committee of the University of Toyama (approval number: 30-99).

This study enrolled 121 patients with recalcitrant plantar warts (single isolated warts in 95 patients and localized mosaic warts in 26 patients). They underwent treatment with a CO_2_ laser with a computerized scanner (AcuPulse, Lumenis Inc) at Toyama University Hospital from 2018 to 2023. The patients were 10-88 years of age (mean: 50.0 years) and healthy. The mean disease duration was 3.1 years (0.4-40 years). They were treated with cryotherapy, salicylic acid vaseline topical therapy, contact immunotherapy, or pulsed dye laser (Table I). After providing their written informed consent, the patients were treated with a CO_2_ laser with a computerized scanner under the same conditions as previously reported after anesthesia.^2^ The details of the setting and method of irradiation are shown in (Supplementary Fig 1, available via Mendeley at https://data.mendeley.com/datasets/6byrbhkn86/1). After irradiation, skin ulcers were treated with gentamicin ointment daily until complete re-epithelization was achieved. We assessed whether the warts had been curatively treated based on the disappearance of black or red dots, which represented thrombosed vessels supplying the wart, on dermoscopic observation at 3 months after irradiation.

**Table I:** Patient baseline characteristics and treatment outcomes

Patient characteristics	Total (%)
Number of patients	121
Sex	
Male	67 (55.0)
Female	54 (45.0)
Age (y)	
Mean (range)	50.0 (10-88)
Duration of illness (y)	
Mean (range)	3.1 (0.4-40)
Previous medical treatment (include duplicates)	
Cryotherapy	121 (100)
Contact immunotherapy	3 (2.5)
Topical active vit D3	11 (9.1)
Topical application of phenol	5 (4.1)
Topical salicylic acid	15 (12.4)
Pulsed dye laser	7 (5.8)
Type of warts	
Simple	95 (79)
Mosaic	26 (21)

Outcome	Total (%)
Evaluation of treatment	
Cure	115 (95)
Recurrence	6 (5)
Time required for epithelialization (d)	
Mean (range)	28.9 (7-70)
Adverse events	
Calluses and scars	12 (10)
Postoperative bleeding	3 (3)

Among the 121 patients, 115 (95.0%) were completely cured after a single treatment session, as shown in (Supplementary Fig 2, available via Mendeley at https://data.mendeley.com/datasets/6byrbhkn86/1). Six patients experienced recurrence within 3 months. Re-epithelialization was completed within 7-70 days (average 28.9 days). Patients who underwent extensive irradiation had a longer time to epithelialization. Calluses and postoperative bleeding occurred as adverse events in 12 and 3 patients, respectively (Table I). The original data are listed in (Supplementary Table I, available via Mendeley at https://data.mendeley.com/datasets/6byrbhkn86/1).

The CO_2_ laser has been used as a laser treatment device for warts since the 1980s.^3^ The remission rate of plantar warts treated with the CO_2_ laser has been reported to vary, ranging from 50% to 100% after 1 or 2 treatment sessions. However, this study showed a high remission rate (95%). For this reason, we hypothesized that the CO_2_ laser with a computerized scanner can evenly vaporize a wart as deep as the upper dermis in the lesion because a continuous focused laser beam moves rapidly across an area. Additionally, the computerized scanner allowed constant irradiation regardless of the practitioner, making the procedure less difficult and providing a stable treatment effect.

One limitation of this study is that the efficacy and safety of this treatment were not compared with other treatments in a control study. Nonetheless, we believe that the present method may be useful for treating recurrent warts.

## Conflicts of interest

None disclosed.
